# Supplementary material for: The Troyer syndrome protein spartin mediates selective autophagy of lipid droplets
Source: Nat Cell Biol. 2023 Jul 13;25(8):1101–10. doi: 10.1038/s41556-023-01178-w (PMC10415183; doi:10.1038/s41556-023-01178-w)
Supplement: Supplementary file 1 — Reporting Summary [file 41556_2023_1178_MOESM1_ESM.pdf]

Corresponding author(s): Tobias C. Walther and Robert V. Farese Jr

Last updated by author(s): May 10, 2023

Reporting Summary

Nature Portfolio wishes to improve the reproducibility of the work that we publish. This form provides structure for consistency and transparency in reporting. For further information on Nature Portfolio policies, see our Editorial Policies and the Editorial Policy Checklist.

Statistics

For all statistical analyses, confirm that the following items are present in the figure legend, table legend, main text, or Methods section.

n/a	Confirmed	
☐	☒	The exact sample size (*n*) for each experimental group/condition, given as a discrete number and unit of measurement
☐	☒	A statement on whether measurements were taken from distinct samples or whether the same sample was measured repeatedly
☐	☒	The statistical test(s) used AND whether they are one- or two-sided<br>*Only common tests should be described solely by name; describe more complex techniques in the Methods section.*
☒	☐	A description of all covariates tested
☐	☒	A description of any assumptions or corrections, such as tests of normality and adjustment for multiple comparisons
☐	☒	A full description of the statistical parameters including central tendency (e.g. means) or other basic estimates (e.g. regression coefficient) AND variation (e.g. standard deviation) or associated estimates of uncertainty (e.g. confidence intervals)
☐	☒	For null hypothesis testing, the test statistic (e.g. *F*, *t*, *r*) with confidence intervals, effect sizes, degrees of freedom and *P* value noted<br>*Give P values as exact values whenever suitable.*
☒	☐	For Bayesian analysis, information on the choice of priors and Markov chain Monte Carlo settings
☒	☐	For hierarchical and complex designs, identification of the appropriate level for tests and full reporting of outcomes
☐	☒	Estimates of effect sizes (e.g. Cohen's *d*, Pearson's *r*), indicating how they were calculated

Our web collection on statistics for biologists contains articles on many of the points above.

Software and code

Policy information about availability of computer code

Data collection	NIS-Elements (4.51.01), IN Cell Analyzer 6000 Acquisition Software v1.0
Data analysis	Lipidomics analyses were done using MaxQuant (1.5.2.8) and Perseus (1.5.1.6). Image analyses were done by open-source software, including Fiji (ImageJ 2.3.0/1.53f51), napari (0.3.4), python (3.7.6), scikit-image (0.19.1), and Cell Profiler (v1.0.9717). The detailed information about imaging analyses for each figure is described in the Method section. Statistic analyses were done by GraphPad Prism 9.

For manuscripts utilizing custom algorithms or software that are central to the research but not yet described in published literature, software must be made available to editors and reviewers. We strongly encourage code deposition in a community repository (e.g. GitHub). See the Nature Portfolio guidelines for submitting code & software for further information.

Data

Policy information about availability of data

All manuscripts must include a data availability statement. This statement should provide the following information, where applicable:

- Accession codes, unique identifiers, or web links for publicly available datasets
- A description of any restrictions on data availability
- For clinical datasets or third party data, please ensure that the statement adheres to our policy

Lipidomics data generated in this study are included in Supplementary Table 3 and 4. Source data have been provided in Source Data. All other data supporting the findings of this study are available from the corresponding authors on reasonable request. Requests will be handled according to the Harvard T. H. Chan School of Public Health policies regarding MTA and related matters.

Field-specific reporting

Please select the one below that is the best fit for your research. If you are not sure, read the appropriate sections before making your selection.

☒ Life sciences ☐ Behavioural & social sciences ☐ Ecological, evolutionary & environmental sciences

For a reference copy of the document with all sections, see nature.com/documents/nr-reporting-summary-flat.pdf

Life sciences study design

All studies must disclose on these points even when the disclosure is negative.

Sample size	Sample size of each measurement was determined by the practical limitations of the protocol utilized. The sample size of Fig. 5f-h was chosen based on the previous studies (Reference 8; PNAS 111, 14924–14929 (2014), 92, 75-83). No statistical methods were used to predetermine sample size. No sample size calculation was performed for fluorescence microscopy experiments. All fluorescence microscopy experiments were repeated 2-3 independent times with more than 5 observations (cells) each.
Data exclusions	No data were excluded for analysis post-image acquisition. Of note, however, for Keima-LiveDrop imaging, cells showing cytosolic soluble Keima accumulation (reflecting a cleavage of Keima fluorescent protein tag from the LiveDrop protein, thereby detaching it from the LiveDrop marker) were not included for image acquisition.
Replication	Number of independent experiments are indicated in the respective figure legends. Replication were successful in all cases.
Randomization	All AAV injection experiments (of Fig. 5f-h) were conducted with randomization of individual animals. For all other cell experiments, randomization was not relevant/not performed, and the control and test conditions were performed on the same day using the same reagents except for the treatment tested (such as RNAi or transfection).
Blinding	All lipidomics and analyses were performed in a blind manner to the AAV genotypes. For all other cell experiments, randomization was not relevant/not performed.

Reporting for specific materials, systems and methods

We require information from authors about some types of materials, experimental systems and methods used in many studies. Here, indicate whether each material, system or method listed is relevant to your study. If you are not sure if a list item applies to your research, read the appropriate section before selecting a response.

Materials & experimental systems

n/a	Involved in the study	
☐	☒	Antibodies
☐	☒	Eukaryotic cell lines
☒	☐	Palaeontology and archaeology
☐	☒	Animals and other organisms
☒	☐	Human research participants
☒	☐	Clinical data
☒	☐	Dual use research of concern

Methods

n/a	Involved in the study	
☒	☐	ChIP-seq
☒	☐	Flow cytometry
☒	☐	MRI-based neuroimaging

Antibodies

Antibodies used	Primary antibodies used in this study were (all primary antibodies are used in 1:1000 dilution): rabbit polyclonal anti-SPG20/spartin (Proteintech, #13791-1-AP), rabbit polyclonal anti-FIP200/RB1CC1 (Proteintech, #17250-1-AP), mouse monoclonal anti-FLAG (Millipore-Sigma, #F3165), anti-FLAG® M2 affinity gel (Millipore-Sigma, #A2220), rat monoclonal anti-HA clone 3F10 (Millipore-Sigma, #11867423001), mouse monoclonal anti-α-tubulin (Millipore-Sigma, #T5168), rabbit polyclonal anti-mCherry (for detection of mScarlet-I, Abcam #ab167453), rabbit polyclonal anti-LC3A/B (Cell Signaling Technology, #4108S), mouse monoclonal anti-actin (Cell Signaling Technology, #3700S), rabbit polyclonal anti-ATGL (Cell Signaling Technology, #2138S), rabbit monoclonal anti-ATG5 (Cell Signaling Technology, #12994T), rabbit monoclonal anti-ATG7 (Cell Signaling Technology, #8558S), chicken polyclonal anti-MAP2 (Synaptic Systems), rat polyclonal anti-GFAP (Thermo Fisher Scientific, #13-030-0), rabbit polyclonal anti-GST (Thermo Fisher Scientific, #A5800).), rabbit polyclonal anti-NBR1 (Proteintech, #16004-1-AP), rabbit polyclonal anti-OPTN (Proteintech, #10837-1-AP), mouse monoclonal anti-SQSTM1 clone 2C11 (Novus, #H00008878-M01). Mouse anti-rabbit IgG-HRP (Santa Cruz Biotechnology, # sc-2357), Mouse anti-IgG kappa binding protein-HRP (Santa Cruz Biotechnology, # sc-516102), Goat anti-rat IgG H&L-HRP (Abcam, # ab97057), DyLight 488-conjugated goat anti-chicken IgY (Thermo Scientific, #SA5-10070), Alexa Fluor 488 donkey anti-rat IgG (Thermo Scientific, #A-21208), and Alexa Fluor 594 goat anti-rabbit IgG (Thermo Scientific, #A-11037).
Validation	Rabbit polyclonal anti-SPG20/spartin (Proteintech, #13791-1-AP; validated with Western blot), rabbit polyclonal anti-FIP200/RB1CC1 (Proteintech, #17250-1-AP; validated with Western blot), mouse monoclonal anti-FLAG (Millipore-Sigma, #F3165; validated with Western blot), anti-FLAG® M2 affinity gel (Millipore-Sigma, #A2220; validated with Western blot), rat monoclonal anti-HA clone 3F10

(Millipore-Sigma, #11867423001; validated with Western blot and immunofluorescence), mouse monoclonal anti-α-tubulin (Millipore-Sigma, #T5168; validated with Western blot), rabbit polyclonal anti-mCherry (for detection of mScarlet-I, Abcam #ab167453; validated with Western blot and immunofluorescence), rabbit polyclonal anti-LC3A/B (Cell Signaling Technology, #4108S; validated with Western blot), mouse monoclonal anti-actin (Cell Signaling Technology, #3700S; validated with Western blot), rabbit polyclonal anti-ATGL (Cell Signaling Technology, #2138S; validated with Western blot), rabbit monoclonal anti-ATG5 (Cell Signaling Technology, #12994T; validated with Western blot), rabbit monoclonal anti-ATG7 (Cell Signaling Technology, #8558S; validated with Western blot), chicken polyclonal anti-MAP2 (Synaptic Systems; validated with immunohistochemistry), rat polyclonal anti-GFAP (Thermo Fisher Scientific, #13-030-0; validated with immunohistochemistry), rabbit polyclonal anti-GST (Thermo Fisher Scientific, #A5800; validated with Western blot), rabbit polyclonal anti-NBR1 (Proteintech, #16004-1-AP; validated with Western blot), rabbit polyclonal anti-OPTN (Proteintech, #10837-1-AP; validated with Western blot), mouse monoclonal anti-SQSTM1 clone 2C11 (Novus, #H00008878-M01; validated with Western blot). Mouse anti-rabbit IgG-HRP (Santa Cruz Biotechnology, # sc-2357; validated with Western blot), Mouse anti-IgG kappa binding protein-HRP (Santa Cruz Biotechnology, # sc-516102; validated with Western blot), Goat anti-rat IgG H&L-HRP (Abcam, # ab97057; validated with Western blot), DyLight 488-conjugated goat anti-chicken IgY (Thermo Scientific, #SA5-10070; validated with immunofluorescence), Alexa Fluor 488 donkey anti-rat IgG (Thermo Scientific, #A-21208; validated with immunofluorescence), and Alexa Fluor 594 goat anti-rabbit IgG (Thermo Scientific, #A-11037; validated with immunofluorescence).

Eukaryotic cell lines

Policy information about cell lines

Cell line source(s)	Human episomal iPSCs (Gibco, Cat# A18944), HEK293T (ATCC, #CRL-3216). SUM159 breast cancer cells were obtained from the laboratory of Dr. Tomas Kirchhausen (Harvard Medical School; original source, RRID:cvcl_5423).
Authentication	We routinely perform cell authentication via STR profiling and the last authentication of the cell lines used in the study was performed on 04/27/22. The generated cell lines were verified with western blot and mass spectrometer-based proteomics.
Mycoplasma contamination	All cell lines tested negative for mycoplasma.
Commonly misidentified lines (See ICLAC register)	No commonly misidentified cell lines were used.

Animals and other organisms

Policy information about studies involving animals; ARRIVE guidelines recommended for reporting animal research

Laboratory animals	C57BL/6J wild-type mice obtained from the Jackson Laboratory (Stock# 000664); male and female randomized; 7- to 10-week-old littermates. All mice were housed on a 12-h light-dark cycle with food and water ad libitum (65±75F and 40±60% humidity).
Wild animals	No wild animals were used in the study.
Field-collected samples	No field-collected samples were used in the study.
Ethics oversight	Studied under the guidelines of the Institutional Animal Care and Use Committee of Wayne State University.

Note that full information on the approval of the study protocol must also be provided in the manuscript.
