# Supplementary figures and images for: The Troyer syndrome protein spartin mediates selective autophagy of lipid droplets
Source: Nat Cell Biol. 2023 Jul 13;25(8):1101–10. doi: 10.1038/s41556-023-01178-w (PMC10415183; doi:10.1038/s41556-023-01178-w)

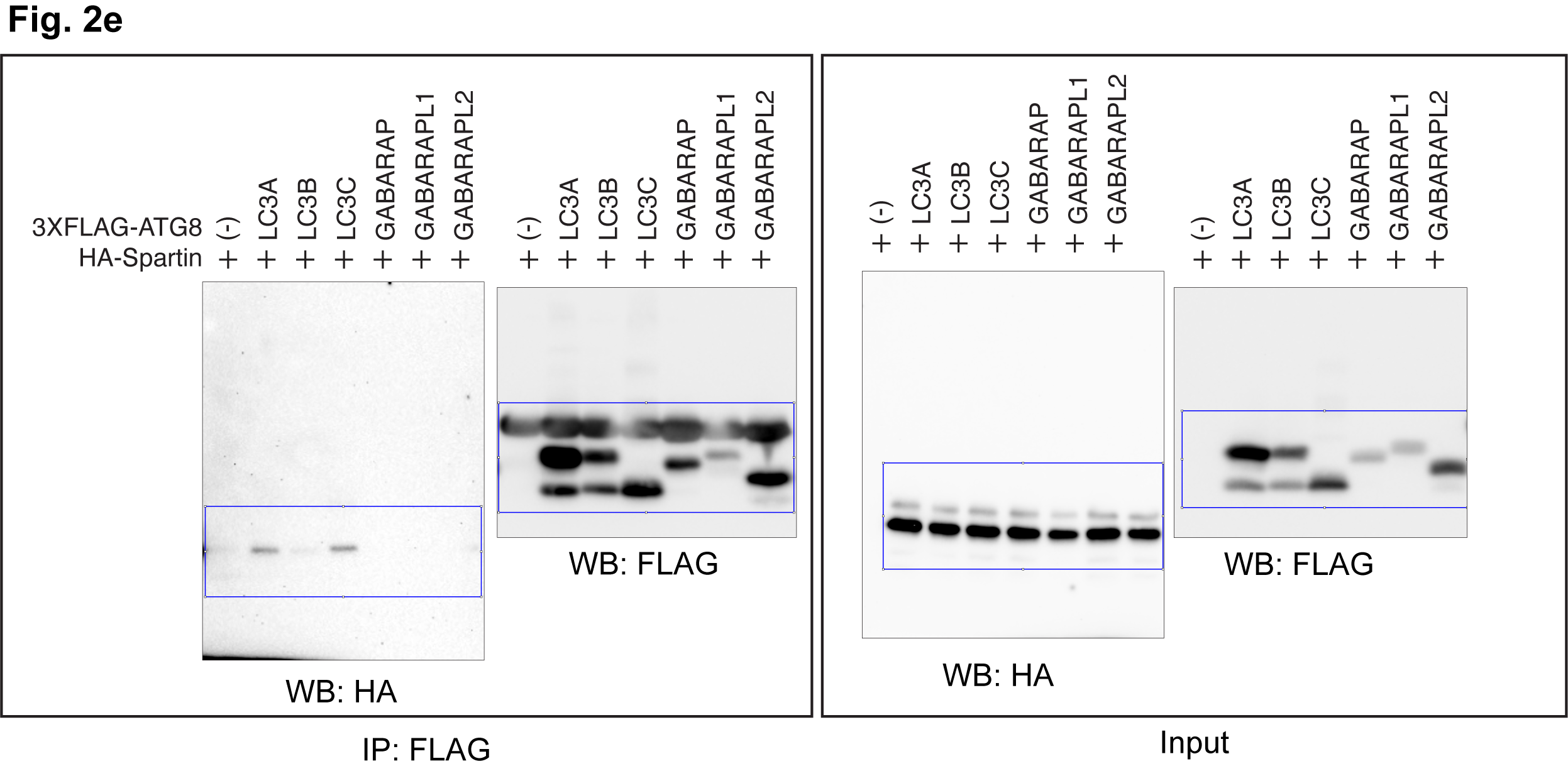

Supplement: Source Data Fig. 2 — Unprocessed WBs. [file 41556_2023_1178_MOESM6_ESM.tif]

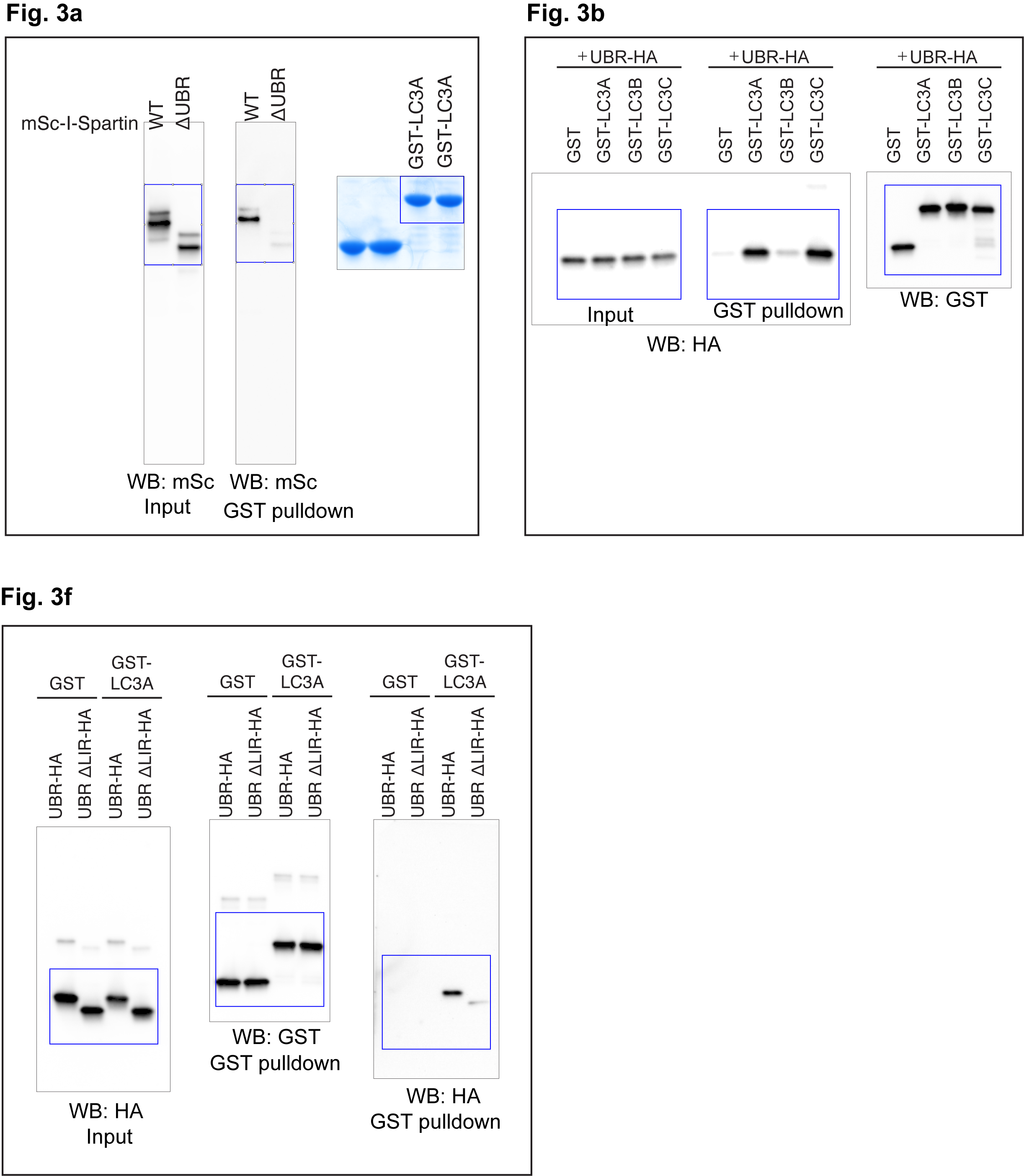

Supplement: Source Data Fig. 3 — Unprocessed WBs. [file 41556_2023_1178_MOESM8_ESM.tif]

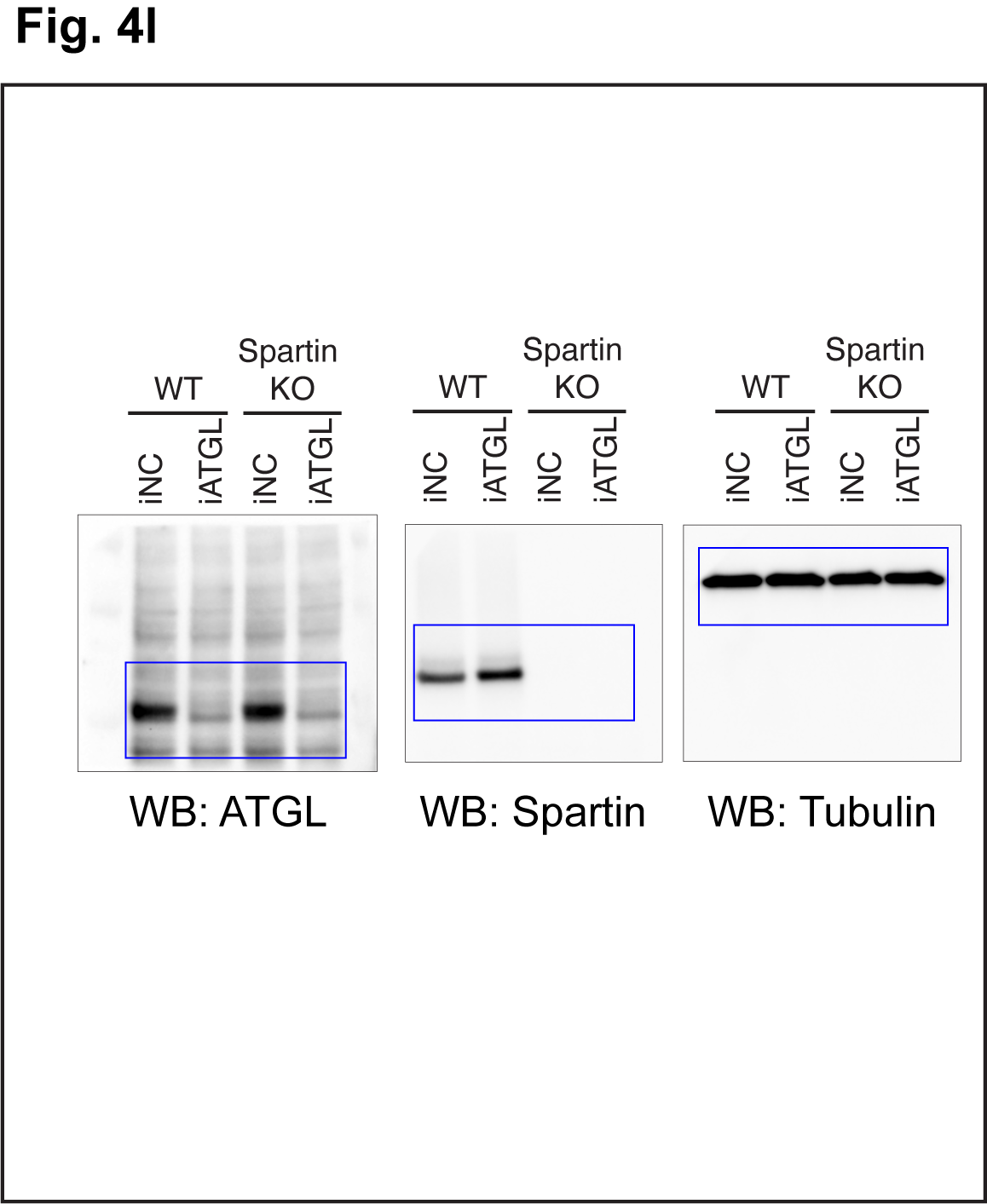

Supplement: Source Data Fig. 4 — Unprocessed WBs. [file 41556_2023_1178_MOESM10_ESM.tif]

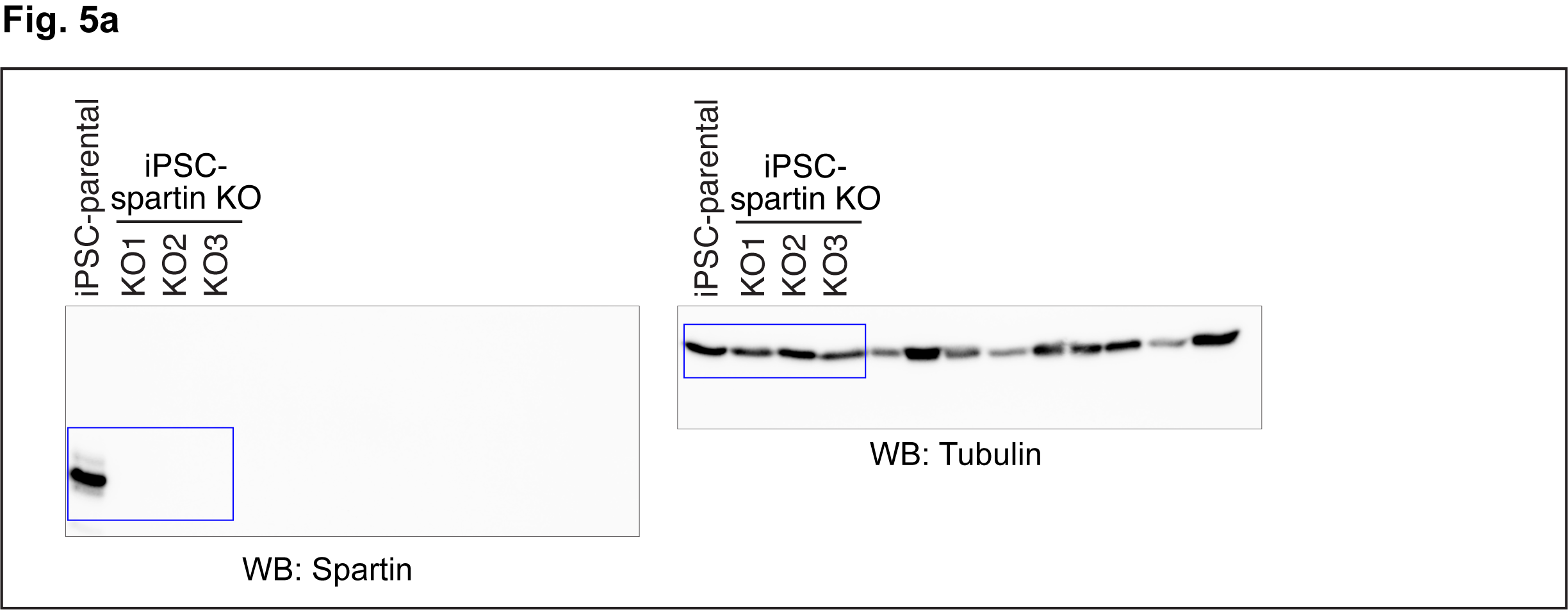

Supplement: Source Data Fig. 5 — Unprocessed WBs. [file 41556_2023_1178_MOESM12_ESM.tif]

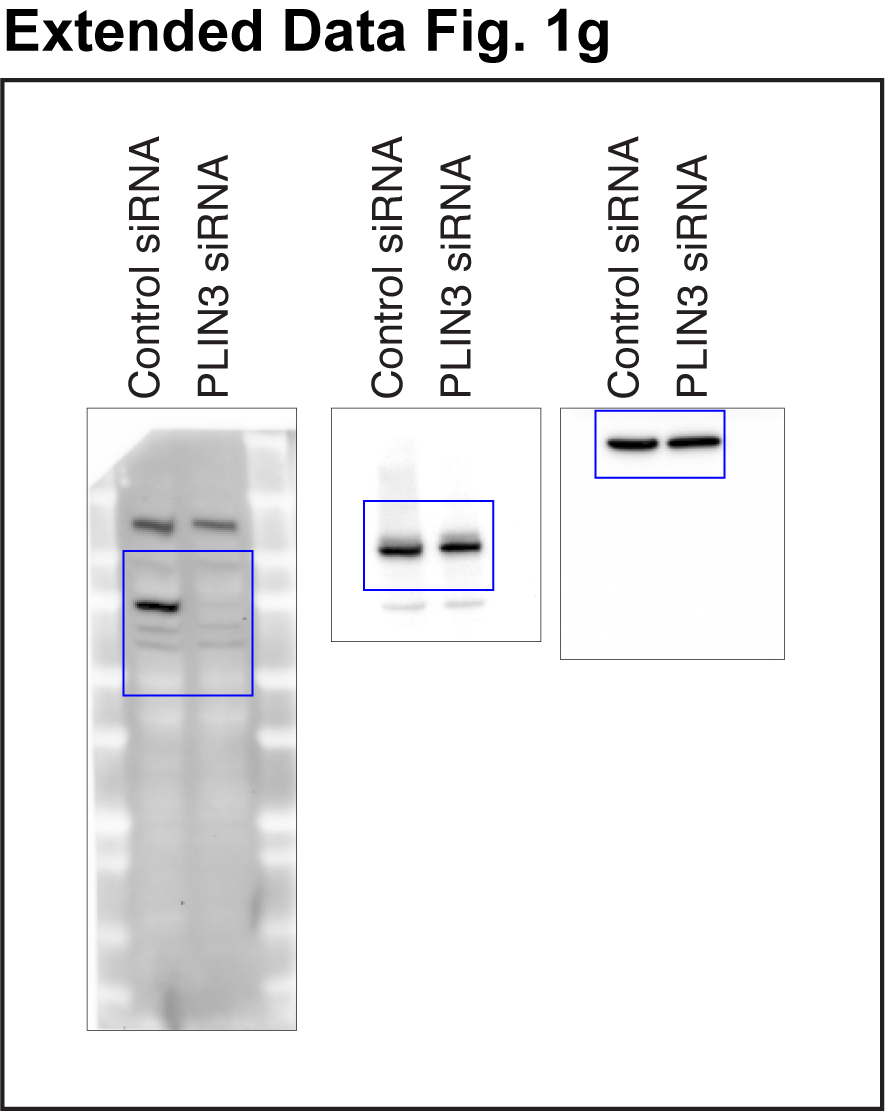

Supplement: Source Data Extended Data Fig. 1 — Unprocessed WBs. [file 41556_2023_1178_MOESM14_ESM.tif]

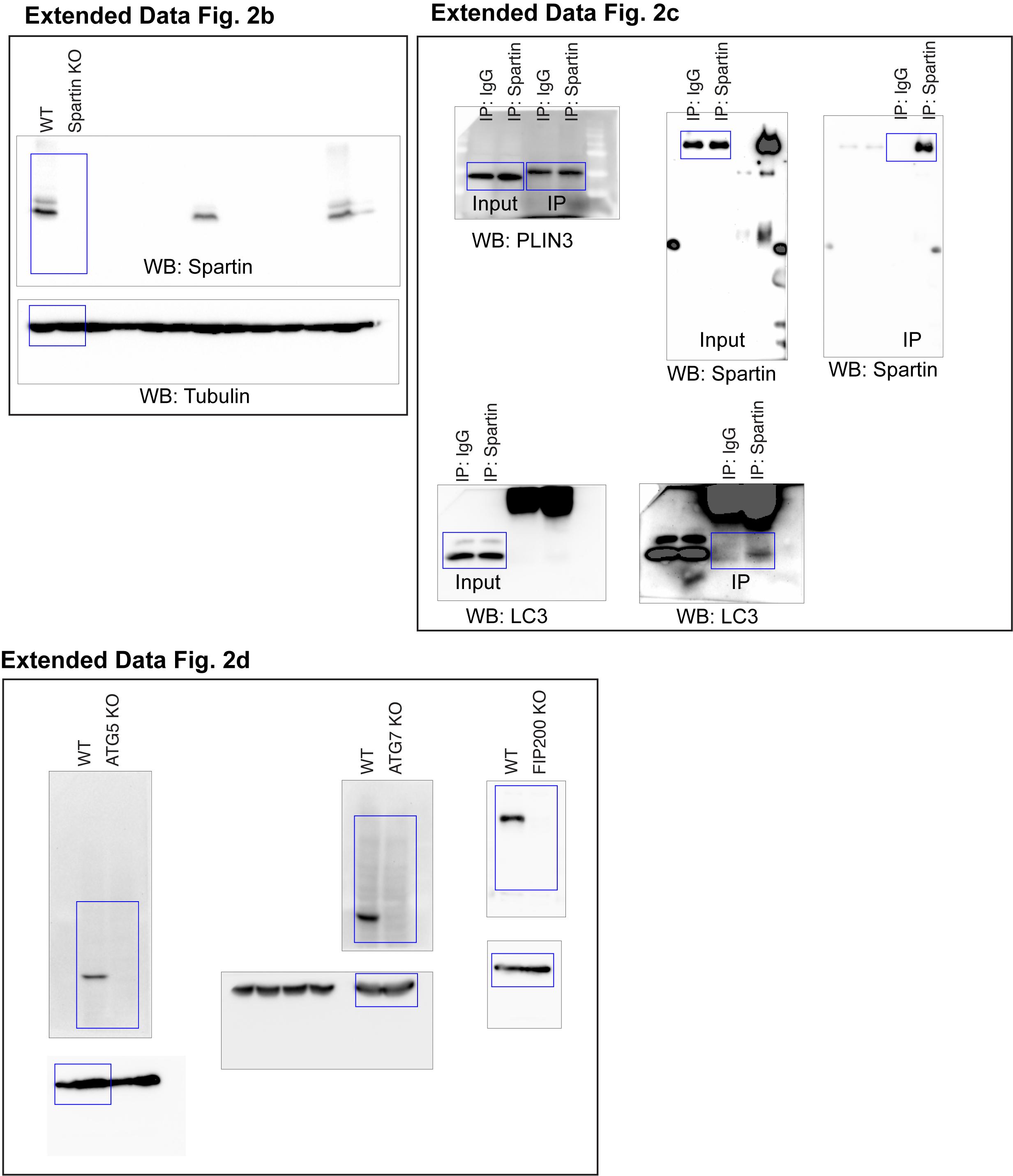

Supplement: Source Data Extended Data Fig. 2 — Unprocessed WBs. [file 41556_2023_1178_MOESM15_ESM.tif]

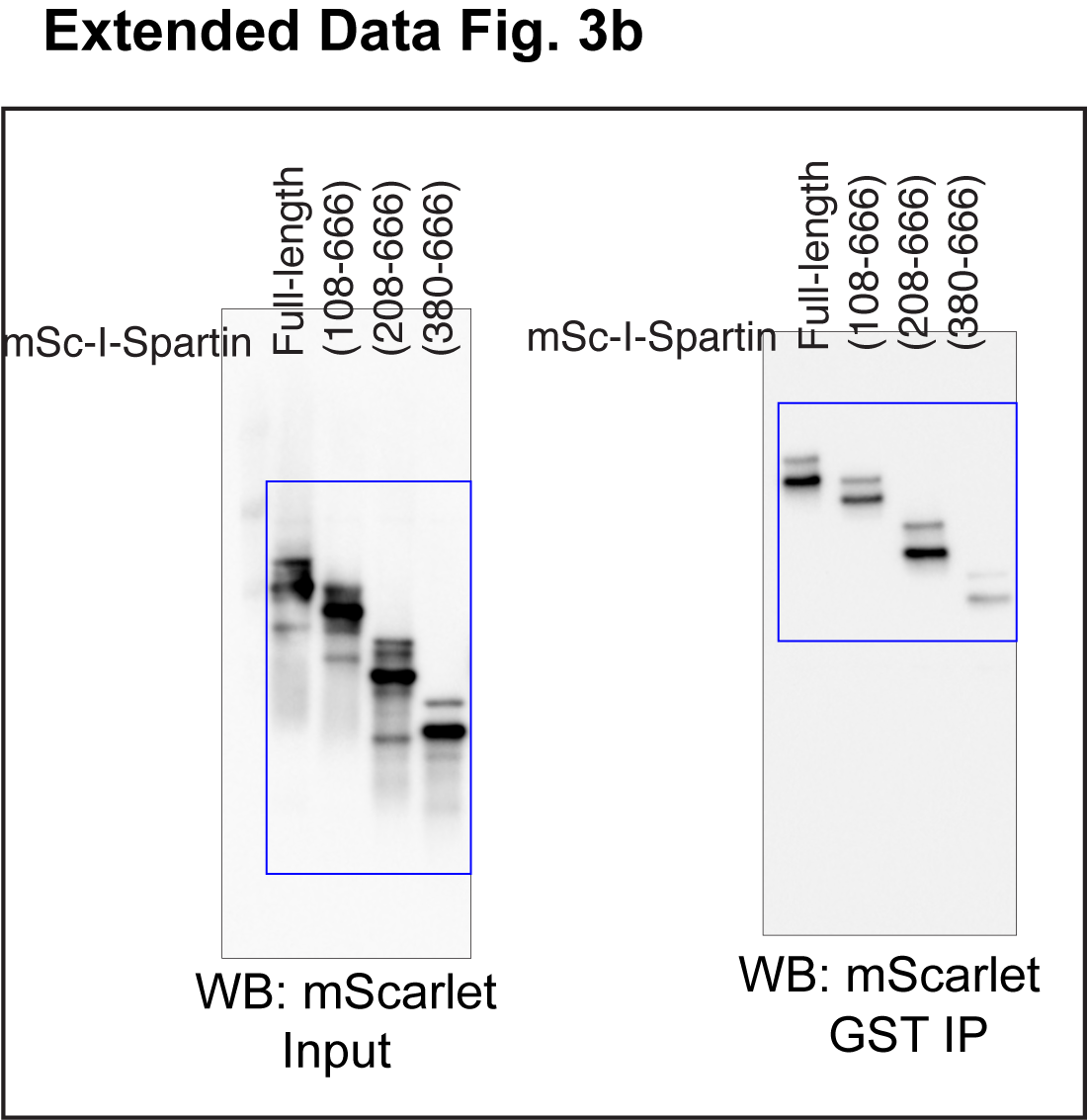

Supplement: Source Data Extended Data Fig. 3 — Unprocessed WBs. [file 41556_2023_1178_MOESM17_ESM.tif]

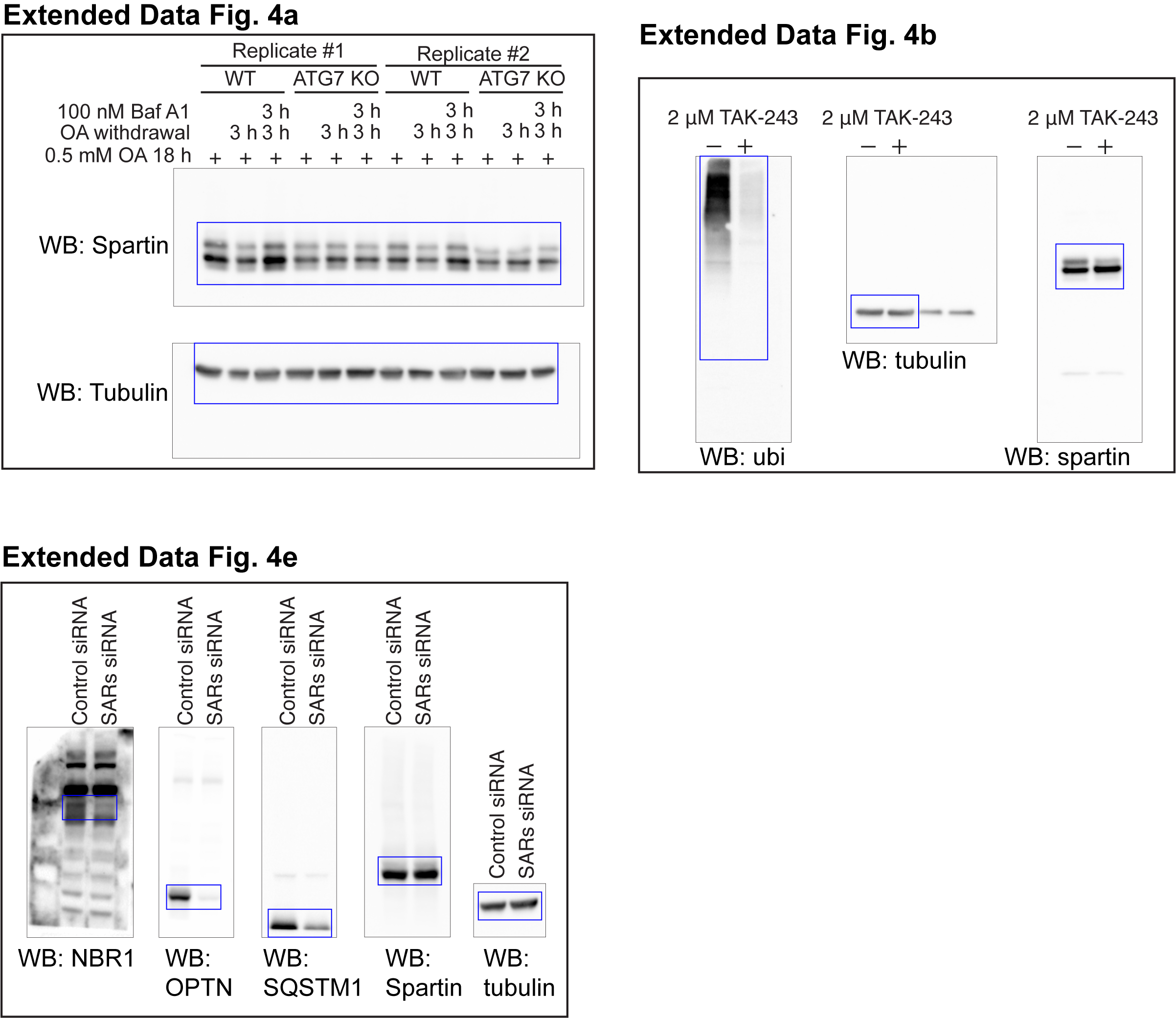

Supplement: Source Data Extended Data Fig. 4 — Unprocessed WBs. [file 41556_2023_1178_MOESM19_ESM.tif]

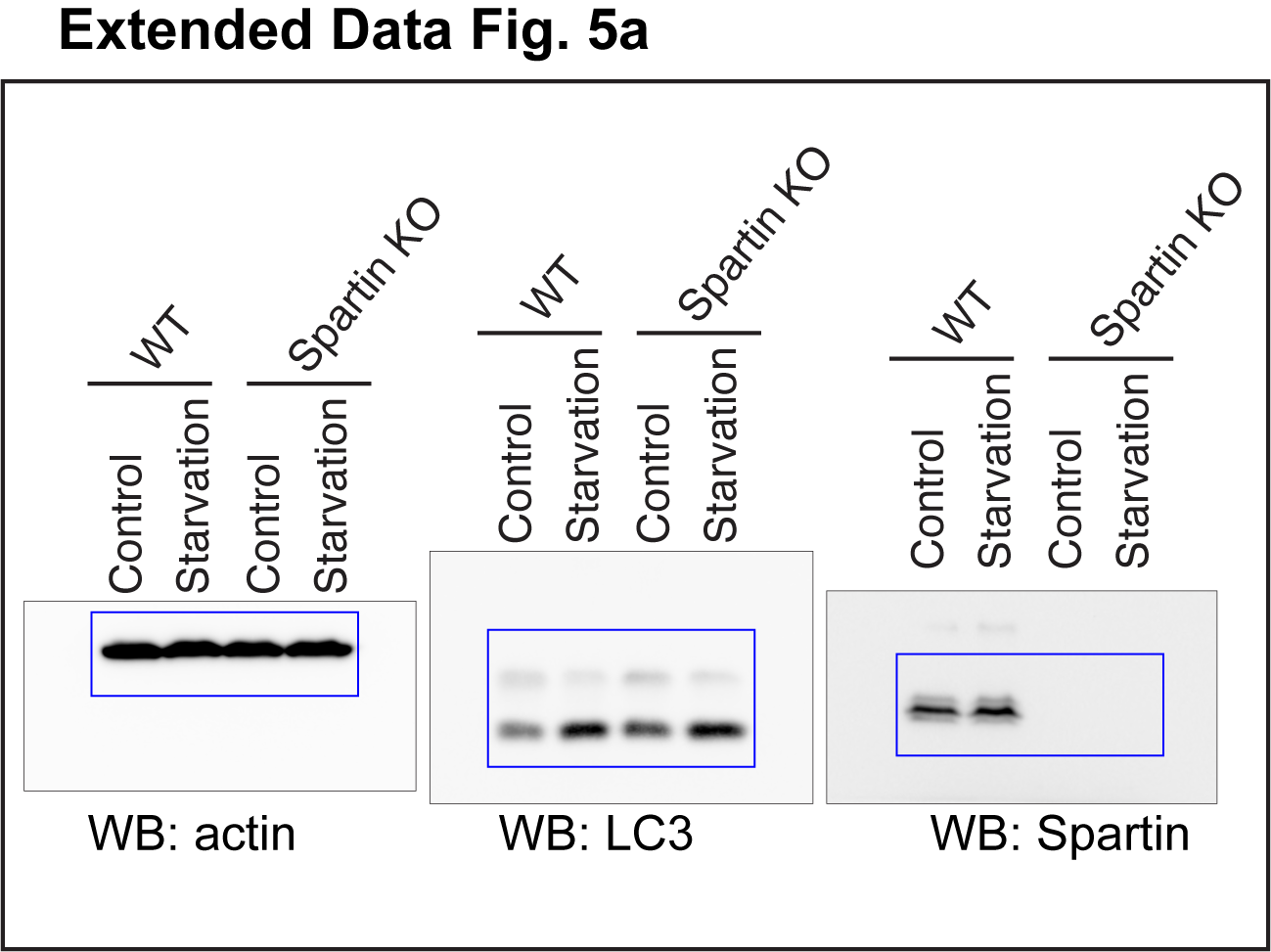

Supplement: Source Data Extended Data Fig. 5 — Unprocessed WBs. [file 41556_2023_1178_MOESM21_ESM.tif]
